# CKD progression, kidney failure, and mortality among US patients with IgA nephropathy

**DOI:** 10.1093/ndt/gfaf084

**Published:** 2025-04-30

**Authors:** John J Sim, Qiaoling Chen, Nancy Cannizzaro, Ancilla W Fernandes, Cibele Pinto, Simran K Bhandari, John Chang, Asher D Schachter, Mohit Mathur

**Affiliations:** Department of Nephrology and Hypertension, Kaiser Permanente Los Angeles Medical Center, Los Angeles, CA, USA; Department of Research & Evaluation, Kaiser Permanente Southern California, Pasadena, CA, USA; Department of Clinical Science, Kaiser Permanente Bernard J. Tyson School of Medicine, Pasadena, CA, USA; Department of Research & Evaluation, Kaiser Permanente Southern California, Pasadena, CA, USA; Department of Research & Evaluation, Kaiser Permanente Southern California, Pasadena, CA, USA; Otsuka Pharmaceutical Development and Commercialization, Inc., Princeton, NJ, USA; Otsuka Pharmaceutical Development and Commercialization, Inc., Princeton, NJ, USA; Department of Clinical Science, Kaiser Permanente Bernard J. Tyson School of Medicine, Pasadena, CA, USA; Department of Internal Medicine, Kaiser Permanente Downey Medical Center, Downey, CA, USA; Department of Research & Evaluation, Kaiser Permanente Southern California, Pasadena, CA, USA; Visterra, Inc., Waltham, MA, USA; Visterra, Inc., Waltham, MA, USA

**Keywords:** epidemiology, IgAN, immunoglobulin A nephropathy, kidney failure

## Abstract

**Background and Hypothesis:**

We assessed disease progression among patients with immunoglobulin A nephropathy (IgAN) and characterized factors associated with risk for adverse outcomes.

**Methods:**

A retrospective longitudinal cohort (2000–2022) study of adults with biopsy-confirmed IgAN within Kaiser Permanente Southern California was performed. The outcome of interest was a composite of ≥50% estimated glomerular filtration rate (eGFR) decline, kidney failure, or mortality. Cox proportional hazards regression modeling was used to estimate hazard ratios (HR) for the eGFR decline/kidney failure with adjustment for potential confounders.

**Results:**

Among 655 patients with primary IgAN (31% Asian/Pacific Islander, 3% Black, 40% Hispanic/Latino, 24% White), 234 (36%) reached the composite outcome of ≥50% eGFR decline (17%), kidney failure (16%), or mortality (3%). The composite outcome occurred at a rate of 79.4 events (95% confidence intervals (CI) 69.6, 90.7) per 1000 patient-years, with a median time to event of 2.7 years. Compared to urine protein creatinine ratio (UPCR) <0.5 vs 0.5–<1 g/g, 1–2, and >2 g/g, the HR (95% CI) for ≥50% eGFR decline/kidney failure were 2.4 (1.1, 5.1), 3.2 (1.5, 6.6), and 5.1 (2.5, 10.4) for baseline UPCR and 5.4 (2.3, 13.0), 14.4 (16.5, 32.2), and 41.2 (17.9, 94.5) for time-averaged UPCR. Lower baseline eGFR and diabetes were also associated with higher risk, while age ≥30 years was associated with lower risk for ≥50% eGFR decline/kidney failure. There were no clear trends differentiating risk by race/ethnicity.

**Conclusion:**

In this large, diverse cohort, high rates of kidney outcomes occurred within a relatively short follow-up duration. Our findings suggest that IgAN carries elevated risk for kidney outcomes starting at proteinuria levels ≥0.5 g/g, in contrast to earlier perceptions that levels below 1 g/g are associated with low risk.

## Graphical Abstract

**Graphical Abstract:**
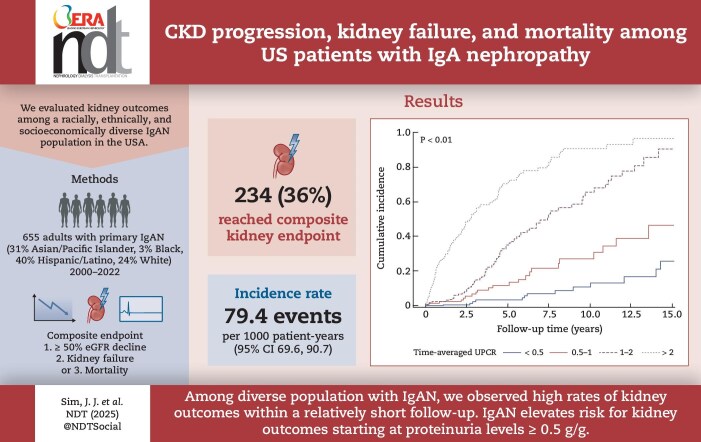

KEY LEARNING POINTS **What was known:**

- Immunoglobulin A nephropathy (IgAN) is a leading cause of chronic kidney disease and has been perceived as a condition that slowly progresses to kidney failure.

**This study adds:**

- To improve understanding of disease progression, we evaluated data from a diverse population enrolled in the Kaiser Permanente Southern California health system who were diagnosed with IgAN by kidney biopsy during 2000–2021.
- We identified 655 adults (mean age 45 years) with primary IgAN, of whom 31% were Asian/Pacific Islander, 3% Black, 40% Hispanic/Latino, 24% White, and 2% other/unknown. A total of 234 (36%) patients reached a composite kidney outcome consisting of ≥50% decline in estimated glomerular filtration rate (*n* = 111; 17%), kidney failure (*n* = 106; 16%), or mortality (*n* = 17; 3%) over a median time of 2.7 years.

**Potential impact:**

- Baseline and time-averaged proteinuria level of ≥0.5 g/g was an important risk factor for disease progression.
- Our data indicate that patients with IgAN may progress faster than previously known.

## INTRODUCTION

Immunoglobulin A nephropathy (IgAN) is one of the most common primary glomerulonephropathies and a leading cause of chronic kidney disease (CKD) [1]. The overall incidence of IgAN has been reported to be at least 2.5 per 100 000 population per year [2], and there is evidence suggesting that incidence has increased over the past few decades [3]. Substantial variation in the epidemiology of IgAN by region has been reported, with prevalence higher in Asia relative to Europe and the USA [3, 4]. We previously reported IgAN incidence rate within Southern California and estimated a US incidence rate of 1.4 per 100 000 population year [4].

The disease is heterogeneous, with variable presentation; clinical manifestations of IgAN range from asymptomatic hematuria or proteinuria to progressive kidney failure [1]. Several risk factors for progression to kidney failure in IgAN have been established, including proteinuria, hypertension, reduced glomerular filtration rate, and MEST-C pathologic score at kidney biopsy [5, 6]. Both the presence and severity of proteinuria are associated with risk for disease progression and mortality [7–9]. The 2021 Kidney Disease: Improving Global Outcomes (KDIGO) glomerulonephritis guidelines designate reduction of proteinuria to <1 g/d as a treatment target in IgAN for improvement of kidney outcomes [10]. This has led to a perception that patients with IgAN and proteinuria <1 g/d are at low risk for progression irrespective of their biopsy findings and would not be eligible for interventions beyond renin-angiotensin-aldosterone system (RAAS) blockade, blood pressure control, and lifestyle management [11]. However, a recent cohort study of patients with IgAN from the UK National Registry of Rare Kidney Diseases (RaDaR) found that 50% reached kidney failure over a median follow up of 5.9 years and hence nearly all could be expected to experience kidney failure in their lifetimes [12]. Of note, high rates of kidney failure within 10 years occurred even in those considered to be at low progression risk [12]. To elucidate whether the reported progression rates are an exception or the norm, additional study of IgAN cohorts is needed in different geographic regions to corroborate the findings from the UK RaDaR analysis.

Accordingly, we evaluated time to kidney disease progression, kidney failure, and mortality within a cohort of racially/ethnically diverse patients with biopsy-confirmed diagnoses of IgAN and treated in a real-world clinical practice environment in the USA, from the Kaiser Permanente Southern California (KPSC) membership. We also investigated patient demographic and clinical characteristics associated with IgAN progression and risk of adverse outcomes.

## MATERIALS AND METHODS

### Study setting and population

A retrospective longitudinal cohort study was performed using data from adult members of KPSC for the period from 1 January 2000 to 30 November 2022. KPSC is an integrated health system with >4.8 million members. The membership is racially, ethnically, and socioeconomically diverse, reflecting the general population of Southern California [13]. Data for study were extracted from electronic health records. This study was approved by the KPSC Institutional Review Board (#5815).

Details of the KPSC glomerulopathy population have been previously described [14–16]. The analysis population for the current study consisted of KPSC members who were diagnosed with IgAN based on kidney biopsies performed between 1 January 2000 and 31 December 2021. For individuals who had multiple biopsies, the first biopsy result was used for inclusion and subsequent analyses in this study. Kidney pathology data was obtained from the KPSC Pathology Database, which captures all KPSC kidney biopsies. The date of the kidney biopsy was used as the index date. Additional inclusion criteria included a minimum of 6 months of continuous membership (allowing for <45-day gaps) in KPSC prior to the kidney biopsy to reliably capture comorbidities.

Exclusion criteria were kidney failure [i.e. estimated glomerular filtration rate (eGFR) <15 ml/min/1.73 m^2^ or treatment with hemodialysis, peritoneal dialysis, or kidney transplant], secondary IgAN, and age <18 years. Secondary IgAN was determined by concurrent diagnosis codes for liver transplant, lupus nephritis, IgA vasculitis, antineutrophilic cytoplasmic antibody vasculitis, hepatitis B, hepatitis C, liver cirrhosis, ulcerative colitis, Crohn's disease, celiac disease, dermatitis herpetiformis, or ankylosing spondylitis [11].

### Comorbidities and clinical measures

All laboratory data, vital sign assessments, and diagnostic and procedure codes are collected in the electronic health record as part of routine clinical care. The presence of comorbidities was assessed based on International Classification of Diseases (ICD) diagnostic coding, medication use, and laboratory or procedure data as appropriate. The Elixhauser Comorbidity Index was determined using ICD diagnosis codes as an overall measure of disease burden. [17] Kidney function was reported as eGFR calculated from serum creatinine levels using the 2021 Chronic Kidney Disease Epidemiology Collaboration Equation (CKD-EPI 2021) [18]. The urine protein to creatinine ratio (UPCR) was presented in g/g. Urine 24-hour protein quantitation was converted to UPCR by dividing the total value by 1000. Urinary albumin-creatinine ratio (UACR) values were converted to UPCR using the equation UPCR = UACR/0.7, and a UPCR value of 0.88 g/g (100 mg/mmol) was considered comparable to protein excretion of 1 g/d when UPCR was not available. The time-weighted averages for UPCR were derived based on UPCR measurements during follow up from kidney biopsy until ≥50% eGFR decline, kidney failure, death, disenrollment from the health plan, or until the end of the study observation period. Time-averaged UPCR was calculated from the area under the curve of serial measurements divided by the length of follow up [12]. A first observation carried back approach was applied if no UPCR was captured at kidney biopsy, and last observation was carried forward as the end of follow-up measurement. To be eligible for time-weighted UPCR calculation, a patient needed to have at least one UPCR measurement during the first 2 years of follow up, and additional values was required if the length of follow up was longer than 3 years.

### Medication utilization

Information on medication use was retrieved from KPSC internal pharmacy dispensing records. Treatment with angiotensin converting enzyme inhibitors (ACE-I), angiotensin receptor blockers (ARB), glucagon-like peptide 1 agonists, and sodium/glucose cotransporter-2 inhibitors (SGLT-2i) was analyzed if dispensed within 1 year prior to kidney biopsy. Treatment with immunosuppressive agents was analyzed if dispensed 4 weeks prior to and up to 1 year following kidney biopsy. Prescriptions <7-day supply or use of immunosuppressive agents that were topical, inhalation, intratympanic, ophthalmic, nebulized, intra-articular, or subconjunctival medications were excluded.

### Race and ethnicity

Race and ethnicity information were entered into the electronic health record based on either patient self-report or provider assessment. Nearly all Hispanics/Latinos within KPSC are Hispanic/Latino White; Hispanic/Latino Black members account for <1% of KPSC. Thus, the designation of Hispanic/Latino within our study refers to Hispanic/Latino White members.

### Study observation period

The date of kidney biopsy was used as index date, and study follow up was censored on 30 November 2022. Individuals were followed until the onset of ≥50% eGFR decline, kidney failure, death, disenrollment from the health plan, or until the end of the study observation period (30 November 2022).

### Outcomes

The primary outcome was a composite outcome of CKD progression (≥50% decline in eGFR), kidney failure (eGFR <15 ml/min/1.73 m^2^, hemodialysis or peritoneal dialysis, or kidney transplant), or all-cause mortality. CKD progression was determined from three eGFR values: the index value, a first eGFR value after the index date with ≥50% decline from index eGFR, and a confirmatory eGFR showing persistent ≥50% decline at ≥30 days from the date of initial ≥50% decline. The eGFR component of kidney failure required a sustained eGFR <15 ml/min/1.73 m^2^ with two values ≥30 days apart. Kidney failure was also evaluated alone as a secondary outcome. For the etiology of mortality, we used the Center for Disease Control categorization for cause of death [19].

### Statistical analyses

Categorical variables are presented as frequencies and percentages. Continuous variables are reported as mean [standard deviation (SD)] or median [interquartile range (IQR)].

Comparisons of demographic characteristics, clinical measures, and comorbidities were conducted for five racial/ethnic groups (Asian/Pacific Islander, Black, Hispanic/Latino, White, and other/unknown). Chi-square or Fisher's exact tests were used for comparison of categorical variables, and one-way analysis of variance or Kruskal–Wallis test for comparison of continuous or ordinal variables.

Both overall and stratified incidence rates of the composite kidney and mortality outcome were calculated per 1000 patient-years. Event rates were calculated for the cohort overall and for each racial/ethnic group. Poisson regression was used to calculate 95% confidence intervals (CI). Cumulative incidence curves were plotted, stratified by UPCR both baseline and time-averaged. The Fine–Gray sub-distribution hazard model was used to estimate hazard ratios (HR) and 95% CI for the composite of 50% eGFR decline or kidney failure, accounting for the competing risk of death. Multivariable HR were calculated with adjustment for potential confounders including age, sex, race/ethnicity, BMI, baseline eGFR, UPCR category, treatment with immunosuppressants, hypertension, diabetes, and hematuria. Proportional hazard assumption was assessed by testing coefficients between transformed time and Schoenfeld residuals. Statistical analyses were conducted using SAS statistical software (version 9.4; SAS Institute, Inc., Cary, NC, USA). Results with *P* < .05 were considered statistically significant.

## RESULTS

### Cohort characteristics

**Figure 1:**
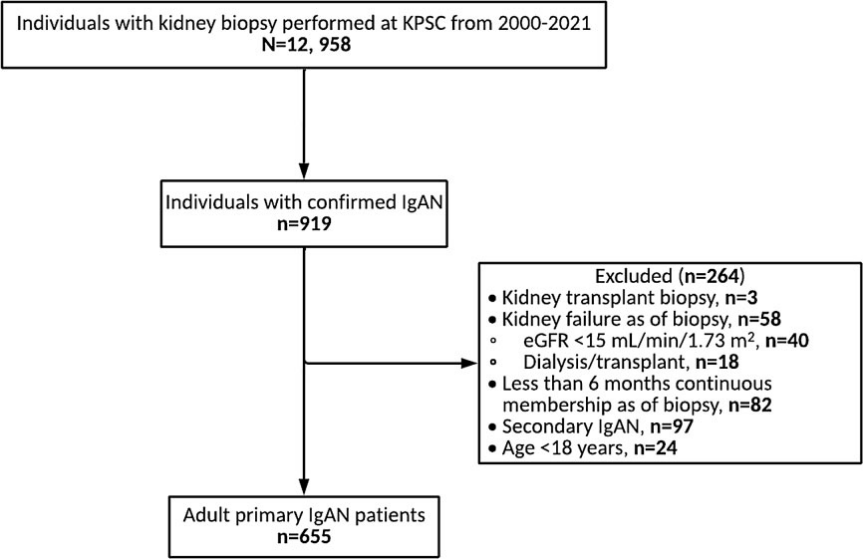
Study population.

Among the 12 958 individuals who underwent kidney biopsy at KPSC during the period 2000–2021, 655 adult patients with primary IgAN met the eligibility criteria for the current analysis (Fig. 1). The mean baseline age of the IgAN cohort was 45.4 years (SD 14.6), with a sex/gender distribution of 48% women and 52% men (Table 1). The racial/ethnic composition of the population was 31% Asian/Pacific Islander, 3% Black, 40% Hispanic/Latino, 24% White, and 2% other/unknown. Patients who were excluded due to ESKD at kidney biopsy were proportionately older and older by mean age (Supplementary Table S1).

**Table 1:** Characteristics of patients with immunoglobulin A nephropathy at biopsy within KPSC (2000–2021).

	Asian/Pacific Islander	Black	Hispanic/Latino	White	Other/unknown	Total	
	(*n* = 201, 30.7%)	(*n* = 20, 3.1%)	(*n* = 259, 39.5%)	(*n* = 159, 24.3%)	(*n* = 16, 2.4%)	(*N* = 655)	*P* value^f^
Age in years at index							.43
Mean (SD)	45.1 (14.0)	45.3 (17.4)	44.7 (14.0)	47.2 (16.4)	39.8 (9.2)	45.4 (14.6)	
18–29, *n* (%)	24 (11.9%)	4 (20.0%)	42 (16.2%)	31 (19.5%)	1 (6.3%)	102 (15.6%)	
30–44, *n* (%)	88 (43.8%)	6 (30.0%)	95 (36.7%)	44 (27.7%)	10 (62.5%)	243 (37.1%)	
45–64, *n* (%)	65 (32.3%)	7 (35.0%)	95 (36.7%)	59 (37.1%)	5 (31.3%)	231 (35.3%)	
≥65, *n* (%)	24 (11.9%)	3 (15.0%)	27 (10.4%)	25 (15.7%)	0 (0.0%)	79 (12.1%)	
Sex, *n* (%)							<.01
Female	118 (58.7%)	8 (40.0%)	128 (49.4%)	51 (32.1%)	6 (37.5%)	311 (47.5%)	
Male	83 (41.3%)	12 (60.0%)	131 (50.6%)	108 (67.9%)	10 (62.5%)	344 (52.5%)	
SBP^a^ in mmHg, mean (SD)	126.5 (14.8)	134.4 (16.4)	129.2 (13.0)	130.2 (13.6)	133.1 (8.3)	128.8 (13.8)	.02
DBP^a^ in mmHg, mean (SD)	77.3 (9.3)	80.0 (11.3)	76.7 (8.9)	76.3 (10.3)	80.6 (7.7)	77.0 (9.4)	.4
BMI in kg/m^2^, mean (SD)	26.3 (4.9)	28.4 (6.3)	30.9 (6.6)	29.5 (6.4)	29.5 (6.1)	29.0 (6.4)	<.01
BMI >30 kg/m^2^, *n* (%)							<.01
No	153 (76.1%)	10 (50.0%)	128 (49.4%)	84 (52.8%)	10 (62.5%)	385 (58.8%)	
Yes	35 (17.4%)	6 (30.0%)	110 (42.5%)	62 (39.0%)	5 (31.3%)	218 (33.3%)	
Unknown	13 (6.5%)	4 (20.0%)	21 (8.1%)	13 (8.2%)	1 (6.3%)	52 (7.9%)	
Smoking status, *n* (%)							.09
Nonsmoker	149 (74.1%)	12 (60.0%)	172 (66.4%)	99 (62.3%)	14 (87.5%)	446 (68.1%)	
Quit smoking	43 (21.4%)	5 (25.0%)	74 (28.6%)	51 (32.1%)	2 (12.5%)	175 (26.7%)	
Current smoker	7 (3.5%)	1 (5.0%)	9 (3.5%)	7 (4.4%)	0 (0.0%)	24 (3.7%)	
Unknown	2 (1.0%)	2 (10.0%)	4 (1.5%)	2 (1.3%)	0 (0.0%)	10 (1.5%)	
Elixhauser Comorbidity Index,^b^ mean (SD)	2.5 (1.8)	2.3 (2.6)	2.9 (1.9)	3.4 (2.8)	2.2 (1.4)	2.9 (2.2)	<.01
Hypertension,^b^ *n* (%)	132 (65.7%)	11 (55.0%)	164 (63.3%)	101 (63.5%)	10 (62.5%)	418 (63.8%)	.91
Diabetes,^b^ *n* (%)	33 (16.4%)	0 (0.0%)	37 (14.3%)	18 (11.3%)	1 (6.3%)	89 (13.6%)	.19
Coronary artery disease,^b^ *n* (%)	8 (4.0%)	0 (0.0%)	4 (1.5%)	10 (6.3%)	0 (0.0%)	22 (3.4%)	.08
Heart failure,^b^ *n* (%)	1 (0.5%)	0 (0.0%)	5 (1.9%)	6 (3.8%)	1 (6.3%)	13 (2.0%)	.15
Stroke,^b^ *n* (%)	3 (1.5%)	0 (0.0%)	1 (0.4%)	4 (2.5%)	0 (0.0%)	8 (1.2%)	.36
Myocardial infarction,^b^ *n* (%)	1 (0.5%)	0 (0.0%)	4 (1.5%)	2 (1.3%)	0 (0.0%)	7 (1.1%)	.74
Atrial fibrillation,^b^ *n* (%)	8 (4.0%)	0 (0.0%)	5 (1.9%)	11 (6.9%)	0 (0.0%)	24 (3.7%)	.08
Peptic ulcer,^b^ *n* (%)	5 (2.5%)	0 (0.0%)	1 (0.4%)	2 (1.3%)	0 (0.0%)	8 (1.2%)	.29
Hematuria,^b^ *n* (%)	92 (45.8%)	14 (70.0%)	123 (47.5%)	90 (56.6%)	8 (50.0%)	327 (49.9%)	.13
Baseline eGFR ^a,c^ in ml/min/1.73 m^2^							
Mean (SD)	61.4 (29.3)	58.5 (24.9)	62.3 (31.6)	54.1 (26.3)	62.1 (28.5)	59.9 (29.5)	
Median (IQR)	54.6 (37.5, 82.5)	63.3 (39.7, 79.5)	56.0 (36.6, 82.8)	49.1 (33.6, 69.6)	59.6 (46.3, 78.4)	53.8 (36.5, 79.6)	.11
≥90, *n* (%)	37 (18.4%)	2 (10.0%)	56 (21.6%)	22 (13.8%)	4 (25.0%)	121 (18.5%)	
60–89, *n* (%)	53 (26.4%)	10 (50.0%)	61 (23.6%)	33 (20.8%)	4 (25.0%)	161 (24.6%)	
45–59, *n* (%)	47 (23.4%)	2 (10.0%)	42 (16.2%)	37 (23.3%)	4 (25.0%)	132 (20.2%)	
30–44, *n* (%)	36 (17.9%)	2 (10.0%)	65 (25.1%)	38 (23.9%)	2 (12.5%)	143 (21.8%)	
15–29, *n* (%)	28 (13.9%)	4 (20.0%)	35 (13.5%)	29 (18.2%)	2 (12.5%)	98 (15.0%)	
Treatment with immunosuppressive agents,^d^ *n* (%)	83 (41.3%)	11 (55.0%)	106 (40.9%)	61 (38.4%)	6 (37.5%)	267 (40.8%)	.71
ACE-I,^b^ *n* (%)	86 (42.8%)	5 (25.0%)	125 (48.3%)	74 (46.5%)	6 (37.5%)	296 (45.2%)	.27
ARB,^b^ *n* (%)	74 (36.8%)	3 (15.0%)	51 (19.7%)	27 (17.0%)	6 (37.5%)	161 (24.6%)	<.01
GLP-1,^b^ *n* (%)	0 (0.0%)	0 (0.0%)	0 (0.0%)	0 (0.0%)	1 (6.3%)	1 (0.2%)	.12
SGLT-2i,^b^ *n* (%)	0 (0.0%)	0 (0.0%)	1 (0.4%)	1 (0.6%)	0 (0.0%)	2 (0.3%)	.75
Baseline UPCR^e^ in g/g							
Mean (SD)	2.7 (2.5)	1.6 (1.9)	2.5 (2.4)	2.2 (2.2)	2.3 (2.3)	2.5 (2.4)	
Median (IQR)	2.0 (1.0, 3.7)	0.8 (0.3, 2.0)	1.8 (1.0, 3.2)	1.6 (0.7, 3.1)	1.5 (0.5, 3.0)	1.8 (0.9, 3.3)	.02
<0.5, *n* (%)	18 (9.0%)	6 (30.0%)	32 (12.4%)	24 (15.1%)	4 (25.0%)	84 (12.8%)	
0.5–<1, *n* (%)	26 (12.9%)	4 (20.0%)	27 (10.4%)	23 (14.5%)	2 (12.5%)	82 (12.5%)	
1–2, *n* (%)	57 (28.4%)	2 (10.0%)	74 (28.6%)	46 (28.9%)	3 (18.8%)	182 (27.8%)	
>2, *n* (%)	95 (47.3%)	5 (25.0%)	111 (42.9%)	54 (34.0%)	6 (37.5%)	271 (41.4%)	
Unknown, *n* (%)	5 (2.5%)	3 (15.0%)	15 (5.8%)	12 (7.5%)	1 (6.3%)	36 (5.5%)	

a Based on the most recent record within 1 year prior to or as of renal biopsy.

b Comorbidity and medication were based on data within 1 year prior to or as of renal biopsy.

c Measurements in the inpatient setting were excluded.

d With immunosuppressive agents during 4 weeks prior to and 1 year after renal biopsy.

e Record measured closest to renal biopsy during 1 year before and 30 days after biopsy was retained. Urine albumin-creatinine ratio and total urine protein within 24 hours were converted to UPCR by dividing by 0.7 and 1000, respectively.

f For comparison of characteristics among the five racial/ethnic groups (Asian/Pacific Islander, Black, Hispanic/Latino, White, and other/unknown).

DBP, diastolic blood pressure; GLP-1, glucagon-like peptide 1 agonist; SBP, systolic blood pressure.

Hypertension was the most frequently occurring comorbidity (64%), and ∼14% of the patients had diabetes. Fifty percent of the patients had hematuria within the previous year. At baseline, the mean eGFR was 59.9 ml/min/1.73 m^2^, and the mean UPCR was 2.5 g/g. A total of 267 (41%) patients received treatment with immunosuppressive therapies (see Supplementary Table S2 for the agents used). Most of the patients were on RAAS inhibitors with 45% receiving ACE-I and 25% receiving ARB (70% of the sample receiving either ACE-I or ARB).

Baseline characteristics were similar among racial/ethnic groups, with a few exceptions (Table 1). There was higher proportion of males represented among Black (60%) and White (68%) patients relative to Asian/Pacific Islander (41%) and Hispanic/Latino (51%) patients. Mean systolic blood pressure was highest in Black individuals (134.4 mmHg) and lowest in Asian individuals (126.5 mmHg), and mean body mass index was highest in Hispanic/Latino individuals (30.9 kg/m^2^) and lowest in Asian/Pacific Islander individuals (26.3 kg/m^2^). Additional significant differences were seen for mean Elixhauser Comorbidity Index (lowest for Black individuals at 2.3 and highest for White individuals at 3.4), use of ARB (least common in Black individuals at 15% and most common in Asians/Pacific Islanders at 37% and other/unknown at 38%), and median UPCR (lowest for Black individuals at 0.8 g/g and highest for Asians/Pacific Islanders at 2.0 g/g).

**Figure 2:**
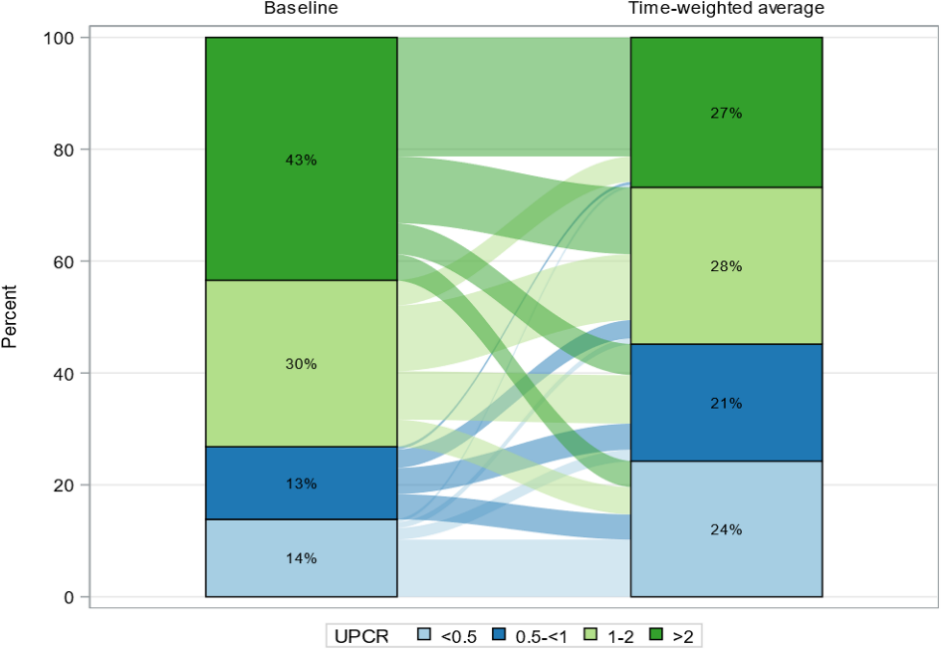
Flow diagram (Alluvial plot) of changes in UPCR at baseline and during follow up.

Over the course of follow up, the distribution of proteinuria changed considerably with time-averaged UPCR compared to baseline UPCR (Table 2). A total of 457 patients had baseline UPCR ≥1 g/g but that declined to 327 patients when assessed with time-averaged UPCR ≥1 g/g. Specifically, 138 patients who had UPCR ≥1 g/g at baseline went on to have time-averaged UPCR ≤1 g/g over the observation period. Similarly, the number of patients with UPCR <1 g/g went from 167 patients with baseline UPCR <1 g/g to 278 patients with time-averaged UPCR <1 g/g. There were 31 patients who had UPCR <1 g/g at baseline who went on to have time-averaged UPCR ≥1 g/g over the observation period (Fig. 2).

**Table 2:** Duration of follow up and time to composite (≥50% eGFR decline, kidney failure, mortality) outcome by patient characteristics.

	Total follow-up time in years	Follow-up time in years [median (IQR)]	Number of events	Incidence rate per 1000 patient-years (95% CI)	*P* value^a^	Time to event in years [median (IQR)]	*P* value^b^	Age at event in years [median (IQR)]
Overall (*N* = 655)	2946	3.1 (1.4, 6.5)	234	79.4 (69.6, 90.7)		2.7 (0.7, 5.5)		49.3 (41.9, 63.2)
Race/ethnicity					.82		.07	
Asian/Pacific Islander (*n* = 201)	951	3.4 (1.6, 6.5)	71	74.7 (58.9, 94.7)		2.9 (1.2, 5.7)		48.0 (41.0, 60.9)
Black (*n* = 20)	70	2.4 (1.5, 3.4)	4	57.5 (18.5, 179.0)		1.0 (0.4, 2.9)		50.9 (34.1, 64.8)
Hispanic/Latino (*n* = 259)	1214	3.3 (1.4, 6.8)	95	78.3 (63.7, 96.2)		2.8 (0.8, 5.8)		46.8 (39.9, 56.3)
White (*n* = 159)	674	3.0 (1.1, 6.5)	61	90.5 (69.5, 117.8)		2.2 (0.5, 4.5)		59.8 (43.3, 71.7)
Other/unknown (*n* = 16)	38	1.6 (0.5, 2.4)	3	79.6 (20.6, 307.5)		0.3 (0.0, 0.5)		47.1 (44.9, 56.2)
Sex					<.01		.62	
Female (*n* = 311)	1456	3.5 (1.5, 7.0)	89	61.1 (49.2, 76.0)		2.4 (0.7, 5.2)		46.1 (39.1, 55.1)
Male (*n* = 344)	1490	2.9 (1.3, 6.1)	145	97.3 (82.3, 115.1)		2.8 (0.7, 5.5)		53.5 (43.0, 67.2)
Age in years					.01		.04	
18–29 (*n* = 102)	410	2.5 (1.0, 6.3)	25	60.9 (41.6, 89.1)		2.9 (1.1, 5.9)		28.4 (26.4, 32.2)
30–44 (*n* = 243)	1155	3.5 (1.5, 7.1)	86	74.5 (60.1, 92.3)		3.1 (0.8, 5.7)		43.3 (36.9, 46.0)
45–64 (*n* = 231)	1103	3.5 (1.7, 6.8)	83	75.3 (60.1, 94.2)		2.4 (0.8, 5.7)		56.9 (51.6, 61.9)
≥65 (*n* = 79)	277	2.4 (0.6, 5.1)	40	144.3 (101.1, 206.1)		1.4 (0.1, 3.3)		75.5 (70.1, 80.7)
BMI					.39		.16	
<30 (*N* = 385)	1645	3.1 (1.5, 6.2)	126	76.6 (64.3, 91.2)		2.9 (0.8, 5.6)		49.3 (38.8, 66.0)
≥30 (*N* = 218)	899	3.1 (1.2, 6.4)	81	90.1 (71.6, 113.3)		2.2 (0.6, 4.4)		50.5 (43.0, 60.4)
Unknown (*N* = 52)	401	4.0 (1.1, 14.6)	27	67.3 (44.3, 102.2)		3.0 (0.8, 10.8)		49.2 (41.7, 60.7)
Treatment with immunosuppressive agents					.03		.11	
No (*n* = 388)	1860	3.2 (1.5, 7.0)	131	70.4 (59.0, 84.1)		2.9 (1.0, 6.0)		47.5 (40.0, 60.4)
Yes (*n* = 267)	1086	3.1 (1.3, 5.9)	103	94.9 (77.7, 115.8)		2.2 (0.5, 4.9)		54.1 (44.1, 67.2)
Baseline eGFR in ml/min/1.73 m^2^					<.01		<.01	
≥90 (*n* = 121)	647	4.2 (1.7, 7.8)	13	20.1 (11.7, 34.6)		3.8 (0.7, 6.3)		33.0 (31.3, 38.5)
60–89 (*n* = 161)	818	4.1 (1.9, 6.8)	38	46.5 (34.3, 62.9)		4.4 (2.8, 7.6)		46.8 (41.2, 56.5)
45–59 (*n* = 132)	606	3.3 (1.5, 6.4)	41	67.7 (51.3, 89.4)		4.3 (2.6, 6.4)		48.2 (41.7, 62.7)
30–44 (*n* = 143)	631	3.0 (1.7, 6.5)	67	106.2 (84.4, 133.6)		2.8 (1.6, 5.6)		54.4 (46.1, 67.2)
15–29 (*n* = 98)	244	1.0 (0.1, 3.2)	75	307.1 (213.0, 442.8)		0.5 (0.1, 2.1)		52.4 (39.1, 67.2)
Baseline UPCR in g/g					<.01		<.01	
<0.5 (*n* = 84)	478	4.5 (2.0, 8.1)	14	29.3 (17.1, 50.2)		2.9 (2.3, 5.4)		70.4 (46.8, 73.8)
0.5–<1 (*n* = 82)	415	3.7 (1.9, 7.0)	19	45.8 (29.6, 70.7)		3.8 (1.6, 7.6)		50.5 (36.4, 61.2)
1–2 (*n* = 182)	844	3.6 (1.6, 7.0)	54	64.0 (49.6, 82.5)		3.9 (2.0, 6.3)		49.8 (44.5, 65.0)
>2 (*n* = 271)	951	2.7 (1.0, 5.1)	130	136.7 (113.8, 164.2)		1.8 (0.5, 4.4)		48.5 (40.0, 60.2)
Unknown (*n* = 36)	257	3.2 (1.1, 12.3)	17	66.1 (40.3, 108.5)		2.2 (0.8, 12.1)		48.9 (38.5, 57.5)
Time-averaged UPCR in g/g					<.01		<.01	
<0.5 (*N* = 150)	1035	6.3 (2.7, 9.7)	13	12.6 (7.4, 21.2)		7.9 (3.0, 12.1)		73.1 (69.6, 78.3)
0.5–<1 (*N* = 126)	671	4.0 (2.2, 7.5)	22	32.8 (22.1, 48.5)		5.4 (2.8, 8.1)		63.1 (41.7, 70.9)
1–2 (*N* = 166)	766	3.9 (2.0, 6.3)	67	87.5 (71.3, 107.2)		4.4 (2.8, 7.0)		50.1 (43.3, 59.8)
>2 (*N* = 159)	430	1.9 (0.7, 3.8)	108	251.1 (206.7, 305.1)		1.6 (0.6, 3.9)		47.1 (36.8, 58.8)
Unknown (*N* = 54)	42	0.3 (0.1, 0.9)	24	566.4 (310.6, 1033.1)		0.1 (0.0, 0.3)		52.9 (42.9, 73.0)
Hypertension					<.01		.3	
No (*n* = 237)	1195	3.6 (1.5, 7.4)	50	41.8 (31.5, 55.5)		3.3 (0.8, 5.9)		43.8 (34.2, 49.4)
Yes (*n* = 418)	1750	3.0 (1.4, 6.1)	184	105.1 (90.6, 122.0)		2.5 (0.6, 5.3)		53.0 (43.3, 66.2)
Diabetes					<.01		<.01	
No (*n* = 566)	2644	3.3 (1.5, 6.8)	191	72.2 (62.5, 83.5)		3.1 (1.1, 5.7)		47.8 (39.5, 58.9)
Yes (*n* = 89)	302	2.4 (0.6, 4.9)	43	142.5 (100.9, 201.1)		1.0 (0.1, 3.0)		63.9 (49.1, 73.8)
Cardiovascular disease/stroke					<.01		.24	
No (*n* = 607)	2755	3.2 (1.4, 6.5)	205	74.4 (64.6, 85.7)		2.8 (0.8, 5.4)		47.1 (39.9, 58.8)
Yes (*n* = 48)	190	2.7 (0.5, 6.0)	29	152.3 (103.6, 223.8)		2.4 (0.1, 5.7)		70.9 (63.9, 75.9)
Hematuria					<.01		<.01	
No (*n* = 328)	1251	2.6 (1.2, 5.4)	153	122.3 (103.5, 144.5)		2.2 (0.6, 4.4)		49.1 (43.3, 62.7)
Yes (*n* = 327)	1694	4.0 (1.7, 7.4)	81	47.8 (38.5, 59.4)		4.1 (1.2, 6.3)		50.9 (39.9, 63.2)

a *P* values are based on robust Poisson regression, comparing incidence rates across different levels.

b *P* values are based on the Kruskal–Wallis test, comparing time to event across different levels.

### Composite outcome of eGFR decline ≥50%, kidney failure, or mortality

A total of 234/655 patients (36%) reached the composite kidney outcome, including either ≥50% decline in eGFR (*n* = 111; 17%), kidney failure (*n* = 106; 16%), or mortality (*n* = 17; 3%) (Supplementary Table S3). The 234 events occurred over an aggregate follow-up time of 2946 patient-years [median per-patient follow up of 3.1 years (IQR 1.4–6.5)], yielding an incidence rate for the composite kidney outcome of 79.4 events (95% CI 69.6–90.7) per 1000 patient-years (Table 2). The median time to composite kidney event during the follow-up period was 2.7 years (IQR 0.7–5.5), and the median age at event was 49.3 years (IQR 41.9–63.2).

No statistically significant differences were noted in the incidences of the composite kidney outcome by race/ethnicity (Table 2). The incidence rate per 1000 patient-years ranged from 57.5 (95% CI 18.5, 179.0) for Black individuals to 90.5 (95% CI 69.5, 117.8) for White individuals. Median time to event was 1.0 years (IQR 0.4–2.9) for Black individuals, 2.2 years (IQR 0.5–4.5) for White individuals, 2.8 years (IQR 0.8–5.8) for Hispanic/Latino individuals, and 2.9 years (IQR 1.2–5.7) for Asian/Pacific Islander individuals. The median age at event was 46.8 years (IQR 39.9–56.3) for Hispanics/Latinos, 48.0 years (IQR 41.0–60.9) for Asians/Pacific Islanders, 50.9 years (IQR 34.1–64.8) for Black individuals, and 59.8 years (IQR 43.3–71.7) for White individuals.

Incidence increased with higher proteinuria levels at baseline and with time-averaged values (Table 2). Using baseline UPCR, incidence per 1000 patient-years was 29.3 (95% CI 17.1, 50.2) among patients with UPCR <0.5 g/g and increased in those with 1–2 g/g [64.0 (95% CI s49.6, 82.5)], with the highest incidence in the group with >2 g/g [136.7 (95% CI 113.8, 165.2)]. Time-averaged UPCR demonstrated greater separation in risk where incidence per 1000 patient-years was 12.6 (95% CI 7.4, 21.2) among patients with UPCR <0.5 g/g, 32.8 (95% CI 22.1, 48.5) in patients with 0.5–<1 g/g, 87.5 (95% CI 71.3, 107.2) in patients with 1–2 g/g, and 251.1 (95% CI 206.7, 305.1) in patients with >2 g/g.

**Figure 3:**
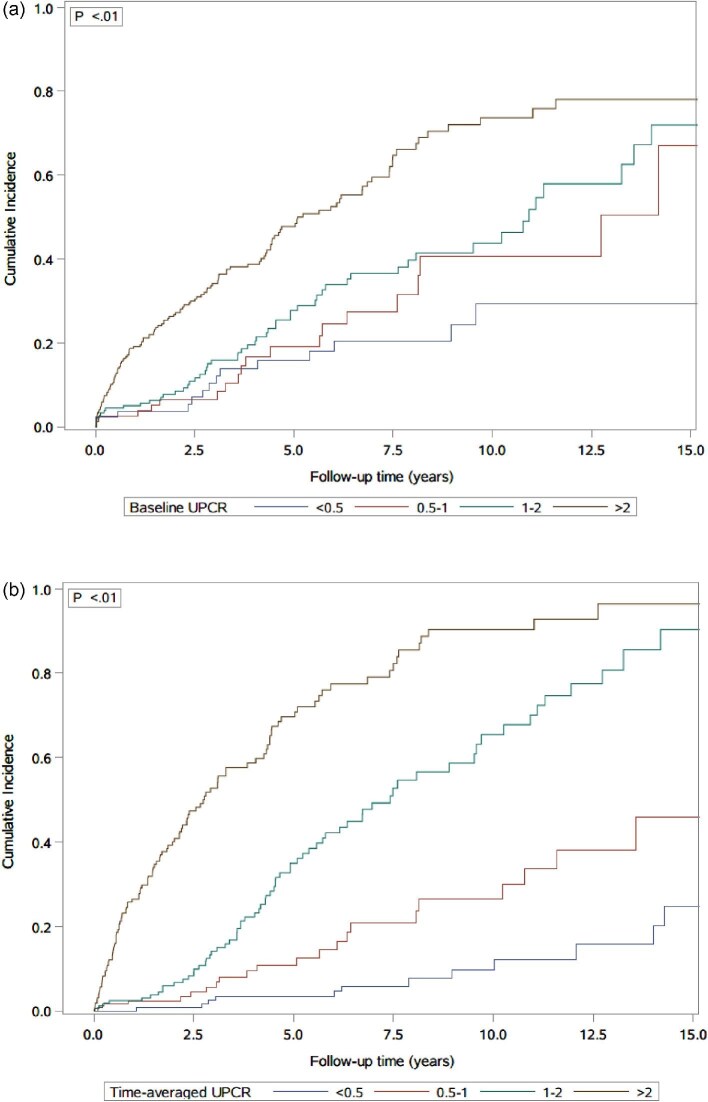
(**a**) Cumulative incidence (incidence risk) of the composite outcome by baseline UPCR. (**b**) Cumulative incidence (incidence risk) of the composite outcome with time-averaged UPCR values.

As UPCR values increased (both baseline or time-averaged), median age at event decreased. For proteinuria at baseline, the proportion of patients who reached the endpoint was 14 of 84 (17%) for those with baseline UPCR <0.5 g/g, 19 of 82 (23%) for those 0.5–<1 g/g, 54 of 182 (30%) for those 1–2 g/g, and 130 of 271 (48%) for those >2 g/g (Table 2). Cumulative incidence (incidence risk) of the composite outcome increased at faster rates in patients with higher baseline and higher time-averaged UPCR with greater separation observed with time-averaged UPCR values (Fig. 3a and b).

The level of kidney impairment at baseline also exhibited a relationship with the composite outcome, whereby incidence per 1000 patient-years was lowest in those with the most preserved kidney function [20.1 (95% CI 11.7, 34.6) at eGFR ≥90 ml/min/1.73 m^2^] and greatest in those with the least kidney function [307.1 (95% CI 213.0, 442.8) at eGFR 15–29 ml/min/1.73 m^2^]. Greater kidney impairment, however, did not consistently exhibit a relationship with median time to event or median age at event (Table 2).

### Regression analyses of composite kidney outcome (eGFR decline ≥50% or kidney failure)

In the multivariable cox proportional hazards regression modeling, lower baseline eGFR, higher UPCR (baseline and time-averaged), younger age, male sex, and the presence of diabetes at baseline were associated with increased hazard of reaching the composite kidney outcome (Tables 3a and b). Using baseline eGFR ≥90 ml/min/1.73 m^2^ as the reference case, the hazard of reaching the composite kidney outcome was >2-fold higher for patients with eGFR 60–89 or 45–59 ml/min/1.73 m^2^, respectively, >5-fold higher for those with eGFR 30–44 ml/min/1.73 m^2^, and 14-fold higher for those with eGFR <30 ml/min/1.73 m^2^. Compared to baseline UPCR <0.5 g/g, the HR (95% CI) for ≥50% eGFR decline/kidney failure were 2.4 (1.1, 5.1), 3.2 (1.5, 6.6), and 5.1 (2.5, 10.4) for baseline UPCR 0.5–<1, 1–2, and >2 g/g, respectively (Table 3a). Compared to time-averaged UPCR <0.5 g/g, the HR (95% CI) for ≥50% eGFR decline/kidney failure were 5.4 (2.3, 13.0), 14.4 (6.5, 32.2), and 41.2 (17.9, 94.5) for time-averaged UPCR 0.5–<1, 1–2, and >2 g/g, respectively (Table 3b). Overall, lower eGFR and diabetes were also associated with higher risk, while age ≥30 years at kidney biopsy was associated with lower risk for >50% eGFR decline/kidney failure. There were no clear trends differentiating risk by race/ethnicity.

**Table 3a:** Multivariable Cox proportional hazards regression modeling to estimate HR and 95% CI for the composite of ≥50% eGFR decline or kidney failure.

Characteristics	Hazard ratio (95% CI)	*P* value
Race/ethnicity (reference = White)		
Asian/Pacific Islander	1.1 (0.7, 1.7)	.73
Black	1.1 (0.3, 4.4)	.84
Hispanic	1.04 (0.7, 1.6)	.84
Other/unknown	1.7 (0.4, 6.5)	.47
Baseline eGFR in ml/min/1.73 m^2^ (reference = 90+)		
60–89	2.4 (1.2, 4.8)	.01
45–59	2.5 (1.3, 5.1)	.01
30–44	5.2 (2.6, 10.5)	<.01
15–29	13.9 (6.6, 29.5)	<.01
Treatment with immunosuppressive agents (yes vs no)	0.7 (0.5, 1)	.07
Baseline UPCR in g/g (reference = UPCR < 0.5)		
0.5–<1	2.4 (1.1, 5.1)	.03
1–2	3.2 (1.5, 6.6)	<.01
>2	5.1 (2.5, 10.4)	<.01
Age in years (reference = 18–29)		
30–64	0.5 (0.3, 0.8)	.01
65+	0.3 (0.1, 0.6)	<.01
Sex (male vs female)	1.3 (0.99, 1.8)	.06
BMI ≥ 30 (yes vs no)	0.96 (0.7, 1.3)	.80
Hypertension (yes vs no)	1.4 (0.95, 2.1)	.09
Diabetes (yes vs no)	1.7 (1.04, 2.7)	.03

*Fine–Gray sub-distribution hazard model used accounting for mortality as competing risk; Patients with unknown UPCR or unknown BMI were included, but results no shown

**Table 3b:** Multivariable Cox proportional hazards regression modeling to estimate HR and 95% CI for the composite of ≥50% eGFR decline or kidney failure using time-averaged UPCR.

Characteristics	Hazard ratio (95% CI)	*P* value
Race/ethnicity (reference = White)		
Asian/Pacific Islander	1.1 (0.7, 1.8)	.55
Black	0.8 (0.1, 4.1)	.76
Hispanic	1.04 (0.7, 1.6)	.85
Other/unknown	0.9 (0.2, 4.4)	.90
Baseline eGFR in ml/min/1.73 m^2^ (reference = 90+)		
60–89	2.5 (1.2, 5.1)	.01
45–59	3.3 (1.6, 6.9)	<.01
30–44	6.7 (3.2, 13.9)	<.01
15–29	15.5 (7, 34.2)	<.01
Treatment with immunosuppressive agents (yes vs no)	0.9 (0.7, 1.2)	.45
Time-averaged UPCR in g/g (reference = UPCR < 0.5)		
0.5–<1	5.4 (2.3, 13)	<.01
1–2	14.4 (6.5, 32.2)	<.01
>2	41.2 (17.9, 94.5)	<.01
Age in years (reference = 18–29)		
30–64	0.7 (0.4, 1.1)	0.09
65+	0.4 (0.2, 0.8)	.01
Sex (male vs female)	0.99 (0.7, 1.4)	.01
BMI ≥ 30 (yes vs no)	0.8 (0.5, 1.1)	.17
Hypertension (yes vs no)	1.3 (0.9, 2)	.20
Diabetes (yes vs no)	1.5 (0.9, 2.5)	.10
Hematuria (yes vs no)	1.0 (0.7, 1.3)	.76

*Fine–Gray sub-distribution hazard model used accounting for mortality as competing risk; Patients with unknown UPCR or unknown BMI were included, but results no shown

### Kidney failure outcome

A total of 179/655 (27%) patients experienced kidney failure (eGFR <15 ml/min/1.73 m^2^, dialysis, or kidney transplant) over an aggregate 3070 patient-years of follow up [median per-patient follow up of 3.3 years (IQR 1.5–7.0)]. The incidence of kidney failure was 58.3 (95% CI 50.1–67.8) per 1000 patient-years (Table 4). The median time to event during follow up was 2.6 years (IQR 0.6–5.7), and the median age at event was 48.8 years (IQR 41.8–61.9).

**Table 4:** Duration of follow up and time to kidney failure (eGFR <15 ml/min/1.73 m^2^, dialysis, or kidney transplant) outcome by patient characteristics (race/ethnicity, baseline eGFR, baseline UPCR).

	Total follow-up time in years	Follow-up time in years [median (IQR)]	Number of events	Incidence rate per 1000 patient-years (95% CI)	*P* value^a^	Time to event in years [median (IQR)]	*P* value^b^	Age at event in years [median (IQR)]
Overall (*N* = 655)	3070	3.3 (1.5, 7.0)	179	58.3 (50.1, 67.8)		2.6 (0.6, 5.7)		48.8 (41.8, 61.9)
Race/ethnicity					.47		.07	
Asian/Pacific Islander (*n* = 201)	1011	3.7 (1.8, 7.1)	57	56.4 (43.3, 73.4)		3.0 (1.5, 6.3)		47.1 (41.8, 60.9)
Black (*n* = 20)	73	2.4 (1.8, 3.4)	1	13.8 (1.7, 110.2)		0.1(0.1, 0.1)		73.1 (73.1, 73.1)
Hispanic/Latino (*n* = 259)	1250	3.5 (1.4, 7.3)	76	60.8 (48.3, 76.6)		2.8 (1.0, 5.6)		46.1 (40.2, 54.5)
White (*n* = 159)	697	2.9 (1.1, 7.0)	42	60.3 (43.7, 83.2)		1.6 (0.4, 4.5)		58.6 (43.7, 69.8)
Other/unknown (*n* = 16)	40	1.7 (0.7, 2.7)	3	75.4 (20.9, 272.1)		0.3 (0.0, 2.6)		49.2 (44.9, 56.2)
Age in years					.1		.01	
18–29 (*n* = 102)	411	2.5 (1.0, 6.5)	15	36.5 (22.1, 60.2)		2.0 (0.4, 6.4)		28.1 (25.3, 33.4)
30–44 (*n* = 243)	1230	3.8 (1.5, 7.6)	73	59.3 (47.0, 74.9)		2.9 (0.9, 6.7)		43.3 (37.6, 45.7)
45–64 (*n* = 231)	1145	3.5 (1.8, 7.2)	67	58.5 (45.6, 75.0)		2.7 (1.1, 6.2)		56.2 (51.3, 62.6)
≥65 (*n* = 79)	283	2.6 (0.6, 5.3)	24	84.7 (53.2, 134.7)		1.0 (0.1, 2.8)		73.0 (69.3, 78.7)
Baseline eGFR in ml/min/1.73 m^2^					<.01		<.01	
≥90 (*n* = 121)	638	4.1 (1.7, 7.8)	5	7.8 (3.2, 19.2)		1.1 (1.1, 1.7)		31.3 (25.3, 34.0)
60–89 (*n* = 161)	899	4.5 (2.0, 7.7)	19	21.1 (13.8, 32.3)		6.7 (4.2, 9.7)		46.1 (42.4, 58.1)
45–59 (*n* = 132)	624	3.3 (1.5, 7.0)	29	46.5 (33.7, 64.1)		5.7 (3.6, 9.6)		45.8 (40.3, 61.8)
30–44 (*n* = 143)	665	3.3 (1.9, 7.1)	57	85.7 (66.1, 111.0)		2.9 (1.9, 5.4)		51.2 (45.5, 62.0)
15–29 (*n* = 98)	244	1.0 (0.1, 3.2)	69	282.6 (194.3, 410.9)		0.5 (0.1, 2.1)		50.9 (38.1, 65.4)
Baseline UPCR in g/g					<.01		.14	
<0.5 (*n* = 84)	489	4.8 (2.0, 8.7)	9	18.4 (9.5, 35.5)		2.9 (2.3, 5.4)		69.8 (46.8, 73.1)
0.5–<1 (*n* = 82)	422	3.8 (1.9, 7.4)	14	33.2 (19.9, 55.4)		4.3 (3.1, 7.6)		49.2 (43.6, 61.8)
1–2 (*n* = 182)	914	3.9 (1.6, 7.8)	40	43.8 (32.2, 59.5)		3.4 (1.2, 6.1)		49.8 (45.0, 66.5)
>2 (*n* = 271)	1017	2.7 (1.1, 5.7)	104	102.2 (83.7, 125.0)		2.0 (0.5, 5.1)		47.0 (40.1, 59.2)
Unknown (*n* = 36)	228	2.6 (0.9, 11.7)	12	52.6 (28.2, 98.3)		1.6 (0.5, 11.1)		47.7 (37.6, 54.4)
Time-averaged UPCR in g/g					<.01		<.01	
<0.5 (*N* = 150)	1043	6.4 (2.7, 10.0)	5	4.8 (2.0, 11.4)		8.9 (6.2, 11.0)		75.6 (47.1, 78.3)
0.5–<1 (*N* = 126)	676	4.1 (2.2, 7.7)	14	20.7 (12.4, 34.5)		4.8 (2.4, 8.1)		64.6 (42.4, 71.0)
1–2 (*N* = 166)	824	4.1 (1.9, 7.1)	53	64.3 (50.6, 81.8)		5.0 (2.9, 7.5)		51.2 (43.7, 60.2)
>2 (*N* = 159)	484	2.2 (0.8, 4.2)	87	179.9 (144.7, 223.5)		2.0 (0.6, 3.9)		46.7 (37.9, 58.2)
Unknown (*N* = 54)	44	0.3 (0.1, 1.1)	20	459.3 (248.3, 849.4)		0.0 (0.0, 0.3)		48.6 (37.5, 72.3)

a *P* values are based on robust Poisson regression, comparing incidence rates across different levels.

b *P* values are based on the Kruskal–Wallis test, comparing time to event across different levels.

Regarding incidence by patient race/ethnicity, the differences were not statistically significant (Table 4). The numerically highest rates of kidney failure per 1000 patient-years were in the categories other/unknown [75.4 (95% CI 20.9, 272.1)] and Hispanic/Latino [60.8 (95% CI 48.3, 76.6)], and the lowest was observed in Black individuals [13.8 (95% CI 1.7, 110.2)]. Median time to kidney failure was shortest among White [1.6 years (IQR 0.4–4.5)] and longest among Asian/Pacific Islander (3.0 years (IQR 1.5–6.3)] individuals. Median age at kidney failure was lowest for Hispanic/Latino individuals [46.1 years (IQR 40.2–54.5)].

As with the composite outcome, incidence of kidney failure per 1000 patient-years increased with increasing baseline UPCR: 18.4 (95% CI 9.5, 35.5) among those with UPCR <0.5 g/g, rising to 33.2 (95% CI 19.9, 55.4) among those with 0.5–<1 g/g, and 43.8 (95% CI 32.2, 59.5) among those with 1–2 g/g (Table 4). Those with UPCR >2 g/g had the highest incidence rate, at 102.2 (95% CI 83.7, 125.0) per 1000 patient-years. Median time to kidney failure was longest among those with baseline UPCR values 0.5–<1 g/g, at 4.3 years (IQR 3.1–7.6), and shortest among those with >2 g/g, at 2.0 years (IQR 0.5–5.1), although the differences were not significant. The median age at kidney failure was 69.8 years (IQR 46.8–73.1) among those with UPCR <0.5 g/g and 47.0 years (IQR 40.1–59.2) among those with UPCR >2 g/g. The differences for kidney failure incidence, median time to kidney failure, and median age at kidney were even greater when assessed across time-averaged UPCR values (Table 4).

The incidence rate of kidney failure increased as baseline eGFR values decreased (Table 4). The incidence rate of those with eGFR ≥90 ml/min/1.73 m^2^ was 7.8 (95% CI 3.2, 19.2) per 1000 patient-years, increasing to 46.5 (95% CI 33.7, 64.1) per 1000 patient-years among those with eGFR in the range of 45–59 ml/min/1.73 m^2^, and nearly doubling among those with eGFR 30–44 ml/min/1.73 m^2^ to 85.7 (95% CI 66.1, 111.0) per 1000 patient-years. At baseline eGFR values 15–29 ml/min/1.73 m^2^, the incidence rate was 282.6 (95% CI 194.3, 410.9) per 1000 patient-years. The relationship of baseline eGFR with time to kidney failure or age at kidney failure did not exhibit a consistent trend.

### Mortality outcome

The overall mortality rate (95% CI) regardless of kidney outcomes was 14.3 (11.0, 18.5) per 1000 patient-years. White patients had the highest incidence at 24.8 (16.3, 37.7). While Asian and Hispanic patients had mortality rates of 10.4 (6.1, 17.7) and 10.4 (6.1, 17.7), respectively (Supplementary Table S4). Overall, only three cases of death were due to infections. The most common cause of death were diseases of the circulatory system, which includes cardiovascular disease (Supplementary Table S5).

## DISCUSSION

We evaluated a racially and ethnically diverse biopsy-confirmed patient cohort with IgAN to determine incidence rates of adverse outcomes and associated risk factors. Kidney-related outcomes (eGFR decline ≥50%, kidney failure, and mortality) occurred in a high proportion of the analysis population (36%) over a median time to event of 2.7 years, with 16% reaching kidney failure during follow up. Although variations in the frequency and time to adverse outcomes were observed by racial/ethnic group, event rates were high across all populations. Historically, IgAN has been regarded as a largely indolent, slow-progressing disease, with ∼30%–40% of patients progressing to kidney failure within 20–30 years of diagnosis and patient subgroups progressing to kidney failure more rapidly [1, 20]. However, this perception is changing due to the results of the UK RaDaR cohort analysis [12]. Our findings are consistent with data from the RaDaR cohort, which demonstrated that 50% of patients with IgAN experienced kidney failure during a median follow up of 5.9 years [12].

Our study also aligns with previous evidence for disease progression among those who had been considered “low risk” (i.e. UPCR 0.5–<1 g/g) [12, 21]. Although higher UPCR was clearly correlated with greater progression risk in the KPSC cohort, substantial percentages of patients with lower baseline UPCR also reached the composite kidney endpoint, including 17% of those with <0.5 g/g and 23% of those 0.5–<1 g/g. What was promising was that the rates of composite outcomes were lower when evaluating across the time-averaged UPCR <0.5 g/g suggesting a benefit of management with protein lowering therapy. These data are consistent with previous observations among Asians indicating improved kidney survival in patients achieving proteinuria <0.5 g/d versus 0.5–<1 g/d [22–24] and suggest the evolving landscape to investigate treatment goals beyond the currently recommended threshold of <1 g/d [10].

Another known risk factor for IgAN progression, kidney function impairment (reduced eGFR), correlated with incidence of the composite kidney outcome and with the kidney failure outcome. Reduced kidney function, however, did not consistently correlate with younger age at event or shorter time to event for either outcome. Age was a confounding variable, with older patients more likely to have reduced eGFR. While the crude rate of kidney outcomes [rate of 60.9 (41.6, 89.1) per 1000 patient-years] was lowest for younger age (18–29 years) patients, multivariable regressions demonstrated that this age group had the highest risk for kidney outcomes. We feel that this finding represents the fact that the younger IgAN patients had less comorbidity burden which were adjusted for in our regressions analyses. It is also possible that younger patients represent a subpopulation with more aggressive disease declaring with clinical markers earlier in life and thus captured and diagnosed early with biopsy. Finally, there may have been some survival bias in our analyses as it appears that the excluded population was on average and proportionately older. Thus, older patients included in our study cohort may have been those with milder disease (Supplementary Table S1).

Taken together, our data underscore the urgency of slowing disease progression and the decline of kidney function in IgAN. Until recently, supportive care has been the primary form of disease management and consists of blood pressure control with the use of RAAS inhibitors (ACE-I and ARB) and SGLT-2i [25, 26]. RAAS inhibitors are used to reduce the rate of loss of eGFR as a first step before initiating corticosteroid therapy [25, 26]. However, evidence for the effectiveness of treatment with corticosteroids is not compelling, and the risk-benefit profile is not optimal, as demonstrated by the TESTING and STOP-IgAN studies [27, 28]. Newer treatments with varied mechanisms of action have been recently approved, and several investigational therapies are in clinical trials [29]. Changing treatment thresholds and emerging precision medicine strategies may become more relevant in advancing care on an individual and population-based level for this potentially devasting disease. In particular, kidney failure imposes a high burden of illness and is a major cost driver in this condition, indicating the need for treatments that inhibit kidney function decline and deterioration in quality of life while reducing mortality [6].

There are potential limitations that may confound the interpretation of our findings. The study cohort was derived from a real-world practice environment and the heterogeneity in management across different providers was reflective of the IgAN treatment landscape. For instance, patients were treated with certain immunosuppression agents such as mycophenolate, azathioprine, or even methotrexate in some cases due to intolerances to steroids, provider interpretation of how aggressive the disease was on biopsy (e.g. crescents), and in consideration of co comorbidities such as rheumatoid arthritis or psoriasis. In addition, we did not have complete and accurate dosing information for medications including ACE-I, ARB, and corticosteroids throughout the observation period. Rather than using a definitive clinical outcome of being treated with dialysis or transplant, we excluded 40 patients categorized as ESKD [30] based on eGFR <15 ml/min/1.73 m^2^ at the time of biopsy. Also, there may be a selection bias, as our IgAN study population may not be representative of all patients within KPSC. Indication for kidney biopsy was determined by individual practitioners, and thus patients with mild IgAN may not have been biopsied and captured in our study cohort. Specific information on histology including mesangial hypercellularity (M), endocapillary hypercellularity (E), segmental glomerulosclerosis (S), and tubular atrophy/interstitial fibrosis (T) and crescents (C) or MEST-C scores were available for only 168 (25%) patients of our cohort (Supplementary Table S6) [31]. Furthermore, our study cohort included patients with comorbidities such as diabetes or hypertension, which in of themselves cause progressive kidney damage and functional decline. We could not account for their impact on the course of IgAN including specific pathology such as diabetic proliferative changes, tubulointerstitial fibrosis, global sclerosis, and hypertensive arteriosclerosis. However, this is again reflective of real-world IgAN patients rather than selective trials or registries. We could not evaluate probable duration of IgAN prior to biopsy date, but the baseline eGFR and proteinuria suggest that our cohort had advanced kidney disease at index. Another example of potential selection bias is our finding pertaining to hematuria. While hematuria is associated with worse kidney outcomes in IgAN [32], we observed that history of hematuria was associated with a lower hazard ratio for the composite outcome. This finding may be related to the fact that those presenting clinically with hematuria (macroscopic or microscopic) may have been identified earlier and/or managed more aggressively. Finally, we may not have captured kidney failure or mortality for patients who experienced these outcomes after disenrollment from KPSC.

Despite these limitations, our findings provide insight into kidney disease progression, kidney failure, and mortality outcomes among a diverse population with biopsy-proven IgAN within a real-world clinical environment. A strength of the study is the inclusion of a large Hispanic/Latino population in addition to Asian/Pacific Islander, Black, and White patients, given that Hispanics/Latinos have been under-represented in prior IgAN research [3]. Higher risk for kidney outcomes was demonstrated starting at UPCR levels as low as 0.5–<1 g/g. Our findings suggest that IgAN is not an indolent disease, and most patients will experience adverse kidney outcomes. More aggressive management strategies with lower thresholds for treatment and that specifically target disease pathophysiology may be the future that could change the course of IgAN.

## Supplementary Material

gfaf084_Supplemental_File

## Data Availability

Individual-level data reported in this study involving human research participants are not publicly shared due to potentially identifying or sensitive patient information. Upon request, and subject to review, KPSC may provide the deidentified aggregate-level data that support the findings of this study. Anonymized data (deidentified data including participant data as applicable) that support the findings of this study may be made available from the investigative team in the following conditions: (i) agreement to collaborate with the study team on all publications, (ii) provision of external funding for administrative and investigator time necessary for this collaboration, (iii) demonstration that the external investigative team is qualified and has documented evidence of training for human subjects protections, and (iv) agreement to abide by the terms outlined in data use agreements between institutions. Interested researchers can submit their request via the Contact Us form (see link https://www.kp-scalresearch.org/aboutus/contact-us/).
