# Supplementary material for: CKD progression, kidney failure, and mortality among US patients with IgA nephropathy
Source: Nephrol Dial Transplant. 2025 Apr 30;40(11):2104–17. doi: 10.1093/ndt/gfaf084 (PMC12559788; doi:10.1093/ndt/gfaf084)
Supplement: gfaf084_Supplemental_File [file gfaf084_supplemental_file.docx]

**SUPPLEMENTARY MATERIAL**

**Supplementary Table S1: Characteristics of Patients excluded who had ESKD at Kidney Biopsy**

|  | **ESKD at biopsy** |  |
| --- | --- | --- |
|  | **(N=58)** |  |
| Age in years at index |  |  |
| Mean (SD) | 49.1 (17.8) |  |
| 18 – 29, n (%) | 8 (13.8%) |  |
| 30 – 44, n (%) | 17 (29.3%) |  |
| 45 – 64, n (%) | 24 (41.4%) |  |
| ≥65, n (%) | 9 (15.5%) |  |
| Sex, n (%) |  |  |
| Female | 22 (37.9%) |  |
| Male | 36 (62.1%) |  |
| Race/ethnicity, n (%) |  |  |
| White | 19 (32.8%) |  |
| Black | 3 (5.2%) |  |
| Hispanic | 22 (37.9%) |  |
| Asian/Pacific Islander | 12 (20.7%) |  |
| Other/Unknown | 2 (3.4%) |  |
| SBP^a^ in mmHg, mean (SD) | 142.1 (13.4) |  |
| DBP^a^ in mmHg, mean (SD) | 81.3 (11.0) |  |
| BMI in kg/m^2^, mean (SD) | 29.7 (7.3) |  |
| BMI >30 kg/m^2^, n (%) |  |  |
| No | 29 (50.0%) |  |
| Yes | 19 (32.8%) |  |
| Unknown | 10 (17.2%) |  |
| Smoking status, n (%) |  |  |
| Nonsmoker | 35 (60.3%) |  |
| Quit smoking | 17 (29.3%) |  |
| Current smoker | 2 (3.4%) |  |
| Unknown | 4 (6.9%) |  |
| Elixhauser Comorbidity Index,^b^ mean (SD) | 4.1 (2.2) |  |
| Hypertension,^b^ n (%) | 40 (69.0%) |  |
| Diabetes,^b^ n (%) | 11 (19.0%) |  |
| Coronary artery disease,^b^ n (%) | 4 (6.9%) |  |
| Heart failure,^b^ n (%) | 9 (15.5%) |  |
| Stroke,^b^ n (%) | 1 (1.7%) |  |
| Myocardial infarction,^b^ n (%) | 1 (1.7%) |  |
| Atrial fibrillation,^b^ n (%) | 4 (6.9%) |  |
| Peptic ulcer,^b^ n (%) | 0 |  |
| Hematuria,^b^ n (%) | 14 (24.1%) |  |
| Baseline eGFR ^a,c^ in mL/min/1.73 m^2^, Median (IQR) | 11.7 (8.6, 14.1) |  |
| Baseline UPCR^e^ in g/g |  |  |
| Mean (SD) | 4.5 (4.2) |  |
| Median (IQR) | 4.3 (2.0, 5.6) |  |
| <0.5, n (%) | 5 (8.6%) |  |
| 0.5 – <1, n (%) | 4 (6.9%) |  |
| 1 – 2, n (%) | 4 (6.9%) |  |
| >2, n (%) | 37 (63.8%) |  |
| Unknown, n (%) | 8(13.8%) |  |
| Treatment with immunosuppressive agents,^d^ n (%) | 31 (53.4%) |  |
| ACE-I,^b^ n (%) | 23 (39.7%) |  |
| ARB,^b^ n (%) | 6 (10.3%) |  |
| GLP-1,^b^ n (%) | 0 |  |
| SGLT-2i,^b^ n (%) | 0 |  |
| ^b^Comorbidity and medication were based on data within 1 year prior to or as of renal biopsy. | | |
| ^c^Measurements in the inpatient setting were excluded. | | |
| ^d^With immunosuppressive agents during 4 weeks prior to and 1 year after renal biopsy. | | |
| ^e^Record measured closest to renal biopsy during 1 year before and 30 days after biopsy was retained. Urine albumin-creatinine ratio and total urine protein within 24 hours were converted to UPCR by dividing by 0.7 and 1000, respectively. | | |
| ^f^For comparison of characteristics among the 5 racial/ethnic groups (Asian/Pacific Islander, Black, Hispanic/Latino, White, and Other/Unknown). | | |

**Supplementary Table S2.** Immunosuppressive agents used in the study cohort

| **Agents** | **Outpatient** | **Inpatient** |
| --- | --- | --- |
|  | **(Number dispensed)** | **(Number of administrations)** |
| **Steroids** |  |  |
| Methylprednisolone | 15 | 294 |
| Dexamethasone | 2 | 90 |
| Prednisone | 890 | 425 |
| Prednisolone | 1 | 0 |
| Budesonide | 0 | 0 |
| **Calcineurin inhibitors** |  |  |
| Tacrolimus | 8 | 0 |
| Cyclosporine | 11 | 0 |
| Voclosporin | 0 | 0 |
| **Alkylating Agent** |  |  |
| Cyclophosphamide | 54 | 94 |
| Chlorambucil | 0 | 0 |
| **Sparsentan** | 0 | 0 |
| **Other** |  |  |
| Sirolimus | 0 | 0 |
| Everolimus | 0 | 0 |
| Azathioprine | 30 | 0 |
| Mycophenolate | 75 | 29 |
| TAVNEOS (Avacopan) | 0 | 0 |
| Methotrexate* | 15 | 0 |
| Belimumab | 0 | 0 |
| Eculizumab | 0 | 0 |
| Obinutuzumab | 0 | 0 |
| Rituximab | 0 | 5 |
| Bortezomib | 0 | 0 |

Outpatient dispenses with less than 7-day of supply were excluded. Topical, inhalation, intratympanic, ophthalmic, nebulization, intra-articular, or subconjunctival medications were excluded. Patient was considered as treated if they had any of the above medication during 4 weeks prior to and up to 1 year after biopsy.

*A total of 4 patients had a history of MTX use; 2 patients for psoriatic arthritis, 1 patient for rheumatoid arthritis, 1 patient was transitioned from prednisone to long term MTX because they had a history of rheumatoid arthritis in the past and was decided with rheumatology.

**Supplementary Table S3.** Study endpoints/outcomes by first outcome

| **Reason for End of Follow-up** | **N (Total=655)** | **%** |
| --- | --- | --- |
| Composite event | 234 | 35.7 |
| Kidney failure | 106 | 16.2 |
| eGFR <15 mL/min/1.73 m^2^ | 90 | 13.7 |
| Transplant/dialysis | 16 | 2.4 |
| eGFR decline ≥50% | 111 | 17.0 |
| Mortality | 17 | 2.6 |
| Disenrollment | 177 | 27.0 |

eGFR, estimated glomerular filtration rate.

**Supplementary Table S4:** Overall mortality outcome

|  | **Total Follow-up Time in Years** | **Follow-up Time in Years  (median [IQR])** | **Number of Events** | **Incidence Rate Per 1000 Patient-Years (95% CI)** | ***P-*value^a^** | **Time to Event in Years (median [IQR])** | ***P-*value^b^** | **Age at Event**  **in Years (median [IQR])** |
| --- | --- | --- | --- | --- | --- | --- | --- | --- |
| Overall (N=655) | 3927 | 4.7 (1.9, 9.1) | 56 | 14.3 (11.0, 18.5) |  | 5.6 (1.2, 10.1) |  | 72.8 (61.3, 79.8) |
| Race/ethnicity |  |  |  |  | 0.1 |  | 0.13 |  |
| Asian/Pacific Islander (n=201) | 1249 | 4.7 (2.3, 9.3) | 13 | 10.4 (6.1, 17.7) |  | 6.9 (3.0, 8.8) |  | 74.3 (65.8, 85.0) |
| Black (n=20) | 73 | 2.4 (1.8, 3.4) | 1 | 13.6 (1.7, 106.6) |  | 0.8 (0.8, 0.8) |  | 73.9 (73.9, 73.9) |
| Hispanic/Latino (n=259) | 1672 | 5.4 (2.0, 10.2) | 20 | 12.0 (7.8, 18.4) |  | 7.0 (2.9, 10.6) |  | 63.4 (53.6, 77.6) |
| White (n=159) | 888 | 4.7 (1.7, 8.2) | 22 | 24.8 (16.3, 37.7) |  | 2.6 (0.6, 7.0) |  | 75.5 (67.3, 80.1) |
| Other/Unknown (n=16) | 44 | 1.8 (1.3, 3.1) | 0 | 0 |  | N/A |  | N/A |

^a^*P*-values are based on robust Poisson regression, comparing incidence rates across different levels.

^b^*P*-values are based on the Kruskal-Wallis test, comparing time to event across different levels.

^c^18 of 60 patients experienced death before >50% eGFR decline / kidney failure

CI, confidence interval; IQR, interquartile range, N/A, not applicable.

**Supplementary Table S5: Distribution of Categorized Cause of Death in the California state death file**

| Primary Cause of Death | Overall mortality (N=56) | |  | Mortality as first outcome (N=17) | |
| --- | --- | --- | --- | --- | --- |
|  | n | % |  | n | % |
| Diseases of the circulatory system | 16 | 28.6 |  | 5 | 29.4 |
| Neoplasms | 9 | 16.1 |  | 3 | 17.7 |
| Endocrine, nutritional and metabolic disease | 8 | 14.3 |  | 2 | 11.8 |
| Diseases of the genitourinary system | 5 | 8.9 |  | 2 | 11.8 |
| Diseases of the digestive system | 4 | 7.1 |  | 1 | 5.9 |
| Certain infectious and parasitic diseases | 3 | 5.4 |  | 1 | 5.9 |
| Diseases of the respiratory system | 3 | 5.4 |  | 1 | 5.9 |
| COVID-19 | 2 | 3.6 |  | 0 | 0 |
| Diseases of the musculoskeletal system and connective tissue | 2 | 3.6 |  | 1 | 5.9 |
| Diseases of the nervous system | 2 | 3.6 |  | 1 | 5.9 |
| External causes of morbidity | 2 | 3.6 |  | 0 | 0 |

Cause of death determined using ICD-10 codes corresponding to CDC-suggested categories); ICD-10, Internal Classification of Disease Tenth Revision.

**Supplementary Table S6: MEST-C scores among IgAN Patients who scores reported (N=168)**

|  | Age | | | | Total  (N=168) |
| --- | --- | --- | --- | --- | --- |
|  | 18-29 (N=19) | 30-44 (N=57) | 45-64 (N=69) | >=65 (N=23) |  |
|  |  |  |  |  |  |
| **M score**, n (%) |  |  |  |  |  |
| m0 | 13 (68.4%) | 34 (59.6%) | 50 (72.5%) | 17 (73.9%) | 114 (67.9%) |
| m1 | 6 (31.6%) | 23 (40.4%) | 19 (27.5%) | 6 (26.1%) | 54 (32.1%) |
|  |  |  |  |  |  |
| **E score**, n (%) |  |  |  |  |  |
| e0 | 5 (26.3%) | 8 (14.0%) | 31 (44.9%) | 9 (39.1%) | 53 (31.5%) |
| e1 | 14 (73.7%) | 49 (86.0%) | 38 (55.1%) | 14 (60.9%) | 115 (68.5%) |
|  |  |  |  |  |  |
| **S score**, n (%) |  |  |  |  |  |
| s0 | 2 (10.5%) | 11 (19.3%) | 17 (24.6%) | 11 (47.8%) | 41 (24.4%) |
| s1 | 17 (89.5%) | 46 (80.7%) | 52 (75.4%) | 12 (52.2%) | 127 (75.6%) |
|  |  |  |  |  |  |
| **T score**, n (%) |  |  |  |  |  |
| t0 | 14 (73.7%) | 37 (64.9%) | 39 (56.5%) | 17 (73.9%) | 107 (63.7%) |
| t1 | 5 (26.3%) | 19 (33.3%) | 29 (42.0%) | 6 (26.1%) | 59 (35.1%) |
| t2 | 0 (0.0%) | 1 (1.8%) | 1 (1.4%) | 0 (0.0%) | 2 (1.2%) |
|  |  |  |  |  |  |
| **C score**, n (%) |  |  |  |  |  |
| c0 | 16 (84.2%) | 43 (75.4%) | 60 (87.0%) | 19 (82.6%) | 138 (82.1%) |
| c1 | 3 (15.8%) | 11 (19.3%) | 8 (11.6%) | 3 (13.0%) | 25 (14.9%) |
| c2 | 0 (0.0%) | 2 (3.5%) | 1 (1.4%) | 0 (0.0%) | 3 (1.8%) |
| Missing | 0 (0.0%) | 1 (1.8%) | 0 (0.0%) | 1 (4.3%) | 2 (1.2%) |
